# A Survey of Crypto Ransomware Attack Detection Methodologies: An Evolving Outlook

**DOI:** 10.3390/s22051837

**Published:** 2022-02-25

**Authors:** Abdullah Alqahtani, Frederick T. Sheldon

**Affiliations:** 1 Department of Computer Science, University of Idaho, Moscow, ID 83843, USA; sheldon@uidaho.edu; 2 Department of Computer Science, Najran University, Najran 61441, Saudi Arabia

**Keywords:** crypto ransomware, data centric, process centric, event-based detection, early detection, deep learning, malware, machine learning-based detection

## Abstract

Recently, ransomware attacks have been among the major threats that target a wide range of Internet and mobile users throughout the world, especially critical cyber physical systems. Due to its unique characteristics, ransomware has attracted the attention of security professionals and researchers toward achieving safer and higher assurance systems that can effectively detect and prevent such attacks. The state-of-the-art crypto ransomware early detection models rely on specific data acquired during the runtime of an attack’s lifecycle. However, the evasive mechanisms that these attacks employ to avoid detection often nullify the solutions that are currently in place. More effort is needed to keep up with an attacks’ momentum to take the current security defenses to the next level. This survey is devoted to exploring and analyzing the state-of-the-art in ransomware attack detection toward facilitating the research community that endeavors to disrupt this very critical and escalating ransomware problem. The focus is on crypto ransomware as the most prevalent, destructive, and challenging variation. The approaches and open issues pertaining to ransomware detection modeling are reviewed to establish recommendations for future research directions and scope.

## 1. Introduction

Ransomware attacks have dramatically increased due primarily to the COVID-19 pandemic that has made people more reliant on computers and online business in what is now called Work from Home (WFH). The attack against Colonial Pipelines that took place in May 2021 is an infamous ransomware incident that disrupted the operations of the major fuel supply chain in 17 states including Washington DC. The company had no choice but to pay around USD 4.4 million. Within the same period, another attack targeted JBS, the world’s largest meat processor. These attacks introduce ransomware to a much broader range of industries than just healthcare, government, and the education sectors. Consequently, these incidents elevated ransomware to the level of a national security concern that prompted the US DOJ to categorize such attacks as terrorist attacks. 

However, the history of ransomware dates back to the late 1990s, when it was forecasted as a potential threat that offensively makes use of cryptography [1,2,3]. Since then, many ransomware variations, or families, have been observed in the wild. Similarly, the severity and evasive characteristics of attack strategies have evolved in response to countermeasure progression [4]. In this regard, ransomware attacks can be distinguished into two categories, crypto ransomware, and locking ransomware. Crypto ransomware is characterized by its ability to encrypt files, thereby immobilizing victims who are thus powerless without the decryption key. Alternatively, locking ransomware locks and/or disables some services in the victim’s machine. This sort of arms race makes current ransomware attacks even harder to handle. While *defenders* identify zero-day vulnerabilities, *threat actors* are busy discovering new ways to break into targeted systems whilst staying undetected for days, weeks or even months.

The efforts of security professionals and researches have converged to fight ransomware attacks [5,6]. They work side-by-side to detect, prevent, and mitigate such attacks and their potential effect. Many research studies investigating ransomware and providing proactive and reactive solutions have been published [7,8,9]. These studies vary based on the focus area, problems they tackle, nature of proposed solutions, and methods they adopt to apply such solutions. As pointed out previously, ransomware is characterized by its penchant to evolve in both intensity and attack strategies. Consequently, this necessitates that developers devote more effort to finding solutions to disrupt this evolution. A comprehensive understanding of existing efforts to supplement the research community in identifying potential opportunities can help to fortify the defensive/protection side of the ecosystem.

The contribution of this survey is four-fold, as follows:

1. Identify and discuss existing research related to crypto ransomware attacks, as the more challenging form of ransomware families.
2. A comprehensive critical analysis of state-of-the-art detection solutions with the focus on the methods, means and techniques used at every phase of the detection model.
3. A focus on existing solutions that adopt machine learning for feature extraction, selection and modeling.
4. Identification of open issues as potential directions for further research endeavors.

In this survey, crypto ransomware and ransomware are used interchangeably, unless mentioned otherwise. The remainder of this survey is organized as follows. Section 2 discusses related work pertinent to ransomware detection. Section 3 discusses the crypto ransomware detection approaches. Solutions for ransomware early detection are discussed in Section 4. Feature extraction, feature selection, and detection techniques are discussed in Section 5, Section 6 and Section 7, respectively. Limitations in existing feature extraction, selection, and modeling techniques were discussed in Section 8, Section 9, Section 10 and Section 11, respectively. Suggestions for research directions are discussed and concluded in Section 12. 

## 2. Related Work

As the incidence of ransomware attacks has increased in recent years, so has the attention of the research community. Numerous studies have investigated different approaches to mitigate threats and their concomitant vulnerabilities. Moreover, several survey papers have been published to summarize the research efforts and provide a more holistic picture concerning the ransomware toxic ecosystem. The review conducted by Al-rimy and Maarof [10] was among the earliest surveys that investigated the factors that contribute to successful ransomware attacks. This review provides a baseline taxonomy for ransomware attacks and characterizes this malicious landscape. Several directions have been also suggested for future research endeavors. 

Another review was conducted by Herrera Silva, Barona López [11] who explored the situational awareness parameters related to ransomware attacks. The authors mapped situational awareness for ransomware into five phases, namely perception, comprehension, projection, decision, and action. Based on these phases, the proposed ransomware detection solutions were evaluated. A taxonomy of Windows-based ransomware detection and prevention solutions was summarized by Moussaileb, Cuppens [12]. These solutions fall under four main categories, namely delivery, deployment, destruction, and dealing. The focus here is on solutions that study user behavior and policies that govern the usage of computer and information technology resources within the organization. Another survey was conducted by Tandon and Nayyar [13], who studied the evolution of ransomware attacks and investigated the architecture of ransomware attacks such as modulus operandi. 

The ransomware detection solutions proposed for mobile systems were explored in the survey conducted by [14]. They approached ransomware issues based on a threat scenario related to the Android environment. Bello, Chiroma [15] investigated the intelligent solutions dedicated to ransomware detection. They focused on those that employ deep learning and big data. Similarly, the survey conducted by Urooj, Al-rimy [16] investigated the detection solutions that had used machine learning with the focus on dynamic analysis. 

From the discussion above, existing surveys pertaining to ransomware-related research put great effort into discussing the different aspect of solutions, ranging from threat modeling, to detection and analysis. They explored the methods and techniques that existing ransomware research used for mitigating the effect on end users. However, the existing surveys did not detail the specifics related to the detection and mitigation process. That is, they overlooked the methodologies that ransomware countermeasures should be based upon, which include the feature extraction, feature selection, and model training/testing. To the best of the authors’ knowledge, there is no survey that investigates ransomware solutions based on the formal phases of detection methodology. To this end, this survey fills this gap by exploring the existing ransomware solutions within the different detection phases, i.e., feature extraction, feature selection, and model training/testing.

## 3. Crypto Ransomware Detection Approaches

It is important to note that several approaches have been explored in the literature that seek to lessen or eliminate the ransomware threat. These efforts can be categorized into prevention and detection. The preventive efforts focus on avoiding damage and/or halting the attack. Several initiatives, such as No More Ransom, have been created to help victims recover from the attacks by decrypting the hijacked files [17,18]. Regarding preventive measures, having regular backup is the golden advice to decrease the damage that ransomware can inflict. However, in many cases, even the backups can become encrypted and decrypting files is quite difficult without having the decryption key. Guessing the decryption key through some form of crypto-analysis, especially for the sophisticated attacks that employ advanced and modern encryption methods, is effectively impossible. 

As mentioned above, relying only on backup is not sufficient as the backup files are also subject to ransomware attacks. If the malware accessed both data and backup files, the backup system would collapse. Therefore, detecting ransomware may be an alternative that could help in identifying the malicious encryption early before it takes place. Numerous strategies to ransomware identification have been developed to counter this threat. Data-centric and process-centric techniques may be subdivided into event-based and machine learning-based approaches, as detailed in the following subsections.

### 3.1. Data Centric-Based Approaches

Data-centric identification aims to track the sources being affected rather than the malicious operation causing the attack [19]. Data-centric crypto ransomware identification techniques [4,5,6,7,8,9] have been explored in several studies. To identify anomalous modifications, most of these solutions offered rely on analyzing user-related documents on a continual basis. Several metrics, in addition to entropy and similarity, are used to attain this objective.

Encryption increases the entropy (degree of disorder) of a file. Therefore, several studies have measured entropy for detecting ransomware attacks [20]. Nolen Scaife et al. [5] used Shannon entropy to quantify the alterations when files are accessed. The proposed method employs a statistical technique to detect any changes to the structure of a user’s files, both before and after access. Additionally, the authors used similarity assessment, which is essentially based on the notion that efficient encryption yields a completely distinct file from its original version. A similar identification procedure was developed by Kharraz et al. [4] by examining the contents of the Input/Output buffer dynamically and quantifying the distinction among the read and written entropy values. Additionally, to identify TorrentLocker ransomware threats that encode the initial portion of the customer’s file, Faustin Mbol et al. [6] leveraged Kullback–Leibler divergence (KBL), also known as the relative entropy metric. Furthermore, Jung and Won [21] leveraged the entropy to measure the change in the file format and determine whether such change has been caused by crypto ransomware attacks.

Another data-centric-based identification method is the decoy procedure used by Moore [11] to identify malicious practices that manipulate consumer documents. Decoy files (also known as honey files) embedded in the user’s machine enable the detection of changes happening to user data as benign programs have no reason to access these files. Similarly, Song et al. [7] recommended analyzing predetermined critical user data locations by embedding decoy files in those locations. To trick ransomware into processing certain files first, Yun Feng et al. [12] located decoy files at the innermost position of every folder so that the detection system raises an alert upon touching those files. Gomez-Hernandez, Alvarez-Gonzalez [22] deployed a set of decoy files into the targeted environment. Once crypto ransomware starts reading these files, its process is blocked. The decoy technique was also utilized by Mehnaz, Mudgerikar [23] to detect and prevent crypto ransomware attacks at the early stage. 

In another study, Morato, Berrueta [20] proposed an algorithm that monitors the traffic specific to shared files for crypto ransomware early detection. Tracking data alone, however, is insufficient proof of maliciousness since comparable modifications may be seen by benign programs that deal with consumer information, such as compression and lawful encryption tools [5,14]. Thus, the decoy approach cannot identify whether the changes were carried out by ransomware, and thus generates many false alarms. Furthermore, ransomware can encrypt the actual data fully or partially before it advances to the decoys [20,24,25]. Moreover, some data-centric measures such as entropy may not be as accurate as expected when the targeted files already have high entropy like the originally-encrypted and/or compressed files [20]. According to Shukla et al. [16], data-centric approaches neglect various access routes that act unpredictably, lowering the detection rate. Furthermore, decoy-based data-centric detection cannot guarantee that the crypto ransomware will attack the decoy files first, which exposes the victim’s data to greater than acceptable risk [20,24]. Therefore, data-centric detection methods are not effective in detecting the ransomware at the early phase of the attack.

### 3.2. Process-Centric-Based Approach

Process-centric-based detection observes the running processes of malicious programs for any suspicious activities or behavior. Such activities could be in the form of certain predefined events related to crypto ransomware, such as generating the encryption key or calling some cryptography-related application programing interfaces (APIs). Those events could be utilized to build event-based detection solutions. Suspicious activities can also be in the form of behavioral patterns observed in the runtime data that malicious processes generate during the run time. These data samples are collected to build a classification model based on machine learning. The following subsections elaborate both event-based detection and machine learning-based detection.

#### 3.2.1. Event-Based Detection

Event-based detection methods search for specific (ad hoc) gauges that indicate an impending crypto ransomware attack. To identify ransomware before it begins its main operation, Ahmadian et al. [17] suggested tracking command-and-control (C&C) traffic to disclose any encoding set, domain generating algorithms (DGA) that provide new domains on demand, and data shared between the infected malware and its distant C&C server. Similarly, Andronio et al. [18] suggested the Heldroid method to track the mechanisms of retrieving threatening messages from the C&C server when it is not typically incorporated in the crypto ransomware payload. In a similar manner, Cabaj, Gregorczyk [26] used Software Defined Networking (SDN) approaches to track http packet patterns and item sizes to discover the cryptoWall class. Furthermore, Le Guernic and Legay [20] presented a strategy for tracking Microsoft’s cryptography APIs, common in many ransomware types, as indications of ransomware attempts to deter it from encrypting the victims’ files. Ad hoc event identification for ransomware has significant disadvantages, since it requires previous knowledge of the encryption technologies used by ransomware, which differ across the families [10,21].

The variation in attack strategies of different crypto ransomware instances gives no guarantee that those aforementioned events will always precede the encryption [27]. Furthermore, independent incidents are insufficient for ransomware identification since they may not happen or may happen at any unexpected time [5,22]. Additionally, outgoing communication tracking may readily be prevented by encrypting such connections [23]. Similarly, today’s sophisticated crypto ransomware can work independently and needs neither an Internet connection nor C&C server assistance [28,29]. Consequently, the events related to the retrieval of encryption key and/or other data may not happen at *any* phase of the attack’s lifetime as these keys could be generated locally the way TeslaCrypt and KillDisk variants do [29]. In addition, these methods have a high likelihood of false alarms since the observed incidents may be disguised [25]. Additionally, most of these incidents are exploited by benign programs and apps, raising the percentage of false alerts [4,20].

#### 3.2.2. Machine Learning-Based Detection

Due to their efficacy in malware detection, machine learning techniques have also been adopted by several crypto ransomware detection studies [28,30,31,32,33,34,35,36,37,38,39,40,41,42,43,44]. These studies employ several classification algorithms to model the behavior of crypto ransomware attacks’ patterns. These classifiers can be distinguished into two categories, single-based and ensemble-based classifiers. Single-based classifiers are individual machine learning classification algorithms, whereas ensemble-based classifiers combine multiple classification algorithms and make them work on the same task complementarily [45,46,47,48]. Single-based classifiers include Support Vector Machines (SVM), Logistic Regression, Decision Tree, and Deep Neural Networks, whereas ensemble-based classifiers include Bagging, AdaBoost and Random Forests. Ensemble learning combines the decisions of multiple single classifiers, also called base classifiers, to produce the final decision. Moreover, the machine learning-based crypto ransomware detection approach can be categorized into delayed detection and early detection as elaborated in the following subsections.

##### Delayed Detection

Delayed detection takes place after observing the entire runtime data generated during the execution of the malicious program [23]. The reason for this is that those detection models are trained using the entire runtime data of each malware instance in the data set. Therefore, the model needs the entire runtime data of crypto ransomware running instance to accurately detect the attack. Malware identification systems, in addition to several crypto ransomware identification studies, make use of this kind of identification technique [25]. The data gathered during the execution of crypto ransomware attempts was utilized by Ahmadian and Shahriari [45] to build a Bayesian Network-based detection model. The data were a mixture of static and dynamic observations. A statistical-based approach to identifying ransomware was presented by Song et al. [7]. The information was acquired by analyzing CPU, I/O, and memory use during crypto ransomware attack execution.

Cusack et al. [30], Alhawi et al. [32], and Cabaj et al. [40] exploited network traffic to obtain information on the data transferred between crypto ransomware operating instances and their C&C servers throughout the attack’s duration. The collected data were used to train machine learning models that were later utilized to detect the attacks based on the observed network traffic in real-time. In their study, Taylor, Smith [49] proposed a crypto ransomware detection model based on data acquired from several physical sensors within the computers during the attacks’ lifetime and used to train a logistic regression classifier. Moreover, Maniath, Ashok [39], Lokuketagoda, Weerakoon [50], Aragorn, YunChun [51] utilized deep learning to build crypto ransomware detection models. Those models were trained with behavioral data captured at the execution time of the malicious software. Cohen and Nissim [35] employed several commonly used machine learning classifiers such as Random Forest (RF), Naïve Bayes (NB), LogitBoost, and Bagging to build several crypto ransomware detection models. The models were trained using data captured from volatile memory throughout several periods of the attacks’ lifetime and recorded into external files. Similarly, Mehnaz, Mudgerikar [23], Daku, Zavarsky [44] utilized the runtime data captured during the execution of ransomware processes to train classifiers based on algorithms such as Naive Bayes (NB), Random Forest (RF), Decision Tree (DT), K-Nearest Neighbors (KNN), and Logistic Regression (LR).

However, delayed detection needs to see the entire data of the running processes of the malware to accurately decide whether it is malicious or benign which, consequently, fails to accurately detect the attack early enough prior to destructive data encryption [23,25,28,40]. This is due to the assumption those studies make about the availability of the entire data at detection time. Accordingly, this assumption builds its detection models based on all data collected from the running malicious process [28,52]. Moreover, delayed detection relies heavily on the post-encryption phase instead of the pre-encryption [23]. Since crypto ransomware attacks are irreversible, such delays in detecting them is ineffective [53].

##### Early Detection

Early identification/prediction [51] aims to identify crypto ransomware threats before they begin encrypting data [20,23,25,28]. As a result, early identification allows for proactive security measures to be taken before the encryption process begins. Das et al. [51] presented a methodology for the early detection of online malware. In the aforementioned research, the identification system used all the information obtained from malware instances throughout its execution process, whereas the Das identification system used just a portion of the information obtained from malware instances during their first 10, 20, 30%, etc., stages. However, the detection accuracy was relatively low, especially when fed only small fractions of data. 

Sgandurra et al. [38] proposed the notion of training machine learning (ML) algorithms for preliminary identification tasks utilizing user information acquired over the early stages of ransomware’s execution period. Their approach uses a fixed time as a threshold by which all ransomware instances in the training dataset are executed for a short amount of time. The threshold was set to 30 s for all crypto ransomware samples. Then, the collected data is used to train a logistic regression classifier. Furthermore, Homayoun, Dehghantanha [40] decreased the threshold down to 10 s for all crypto ransomware samples. Then, a pattern mining technique was proposed to extract the features by which the detection model was built using several ML techniques including Decision Tree and Bagging. Later, in another study, Homayoun, Dehghantanha [31] followed the same fixed-thresholding approach set at 10 s to train a deep learning model for ransomware early detection. Similarly, Rhode, Burnap [28] employed the same approach with a threshold fixed to one second for all crypto ransomware instances. Subsequently, the extracted data were used to train multiple ML classifiers such as AdaBoost, RNN and Random Forest.

Despite the importance of using early data from ransomware attacks for training detection models, previous studies extracted early data based on a fixed time for all instances, which is unsuitable given the non-stationary (dynamic) nature of crypto ransomware behavior [27,33,44]. In other words, the constant thresholding may ignore the encryption initiation place in many cases, resulting in the collected data not correctly representing the initial stage of the attack when encryption has not yet occurred. Thus, early detection model may not detect the ransomware *before* encryption. Moreover, the early data contain incomplete attack patterns. Unfortunately, such insufficiency in attack patterns has a negative impact on the process of features extraction, selection, and model training [28,54,55].

## 4. Related Techniques for Building Early Detection Models

Due to the irrecoverable nature of crypto ransomware attacks, it is critical to identify them early on, prior to encryption. To detect such attacks as early as possible, existing research has proposed several models. Those models are composed of several components, each of which employs one or more techniques. Within each component, one or more techniques have been used to achieve better detection accuracy and reduce false alarms.

**Figure 1:**
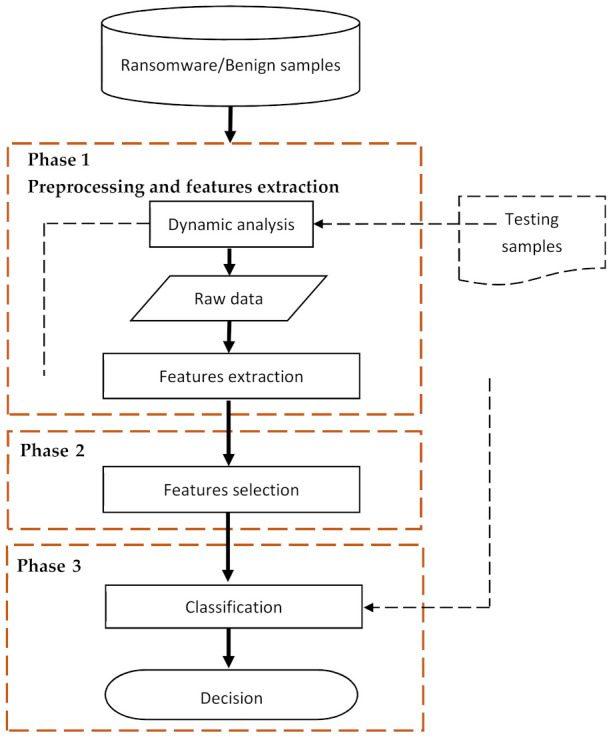
Generic design of crypto ransomware early detection models.

According to the existing literature, the early detection models start by extracting and selecting the discriminating features, which then are used to train the early detection model [28,31,40,42]. During feature extraction, the features are extracted from crypto ransomware runtime data, which can be either numerical or textual. In the case of textual data, feature extraction involves tokenization by which the textual data are transformed into the numerical form. Several techniques can be employed to carry out such tokenization, including Bag of Words (BoW), Term Frequency (TF), and Term Frequency-Inverse Documents Frequency (TF-IDF) [56]. During feature selection, a subset of the informative features is selected to decrease data dimensionality and prevent overfitting. According to existing works, several feature selection techniques have been used, such as information theory-based, statistical-based, and similarity-based techniques [57]. On model training/testing, a machine learning classifier is trained using the data and features extracted and selected in the previous phases. Several machine learning classifiers have been involved in existing works to build and train the detection model, including Support Vector Machine (SVM), Decision Tree, and Logistic Regression, in addition to several ensemble-based classifiers [28,42]. Figure 1 shows the generic design of crypto ransomware early detection models according to the related works described above. This figure shows that the generic model has three components, each of which corresponds to its own phase. In particular, pre-processing and feature selection belong to phase 1; feature selection belongs to phase 2; and training/testing belongs to phase three. The following subsections elaborate the techniques used in each of those components.

## 5. Feature Extraction Techniques

In crypto ransomware and malware detection, feature extraction techniques extract patterns that represent the behavior of malicious programs from the runtime data of those malicious codes. As the crypto ransomware runtime data are stored in the trace files in a textual form, those trace files can be viewed as documents and API calls in those files can be seen as terms (words) [58]. Accordingly, several malware and crypto ransomware detection studies utilized the features extraction techniques used in text mining, such as vectorization, to transform the textual data into numerical form and also extract the attacks’ features [30,31,43,59,60,61,62]. Based on the analysis type, these features can be structural (static) or behavioral (dynamic) [30,33,63,64]. That is, structural features come from static analysis whereas behavioral features come from dynamic analysis [30,31,65]. Although both types were employed for malicious software detection, the early detection evokes the usage of behavioral features dynamically extracted from the runtime data. 

The Bag of Words (BoW) is an n-gram-based features extraction method utilized by many text and data mining applications [66]. It is also leveraged by many malware detection studies to group N consecutive API calls as one feature [60,67,68,69]. For crypto ransomware detection, the n-gram technique has been employed by Sgandurra, Muñoz-González [42] to build the API-based feature set. The technique was also used by Chen and Bridges [43] to extract the features that represent WannaCry crypto ransomware attacks. Similarly, Zhang, Xiao [30] utilized n-gram to extract the crypto ransomware features from opcode sequences. Similarly, Homayoun, Dehghantanha [31] employed N-gram to transform the sequence of crypto-ransomware events stored as a text into numerical values. It is worth mentioning that N-gram is usually coupled with word frequency techniques such as Term Frequency (TF) to count the number of times the word (term) has occurred in a particular trace file [70]. However, TF treats each term (API in crypto-ransomware case) equally and does not distinguish between the common (general purpose) APIs from those closely related to the attacks [56].

Term Frequency-Inverse Document Frequency (TF-IDF) addresses the limitations of the TF technique and distinguishes between general purpose and attack-specific API calls [56]. It downweights the APIs used by all samples of both benign and ransomware programs, as they are considered to be generic APIs that have no specific information about the attack. When the number of instances containing a specific feature $x_{q}$ increases, the denominator of the expression increases, and consequently the feature value $\omega \left(x_{q}\right)$ decreases. TF-IDF is calculated according to Equation (1) as follows:

(1) $$\omega^{\;}\left(x_{q}\right)=tf^{\;}\left(x_{q}\right)\cdot \log\frac{N}{idf\left(x\right)+1}$$

where $w\left(x_{q}\right)$ denotes the TF-IDF weight of feature x in the instance q; $tf\left(x_{q}\right)$ denotes the frequency of x in the instance q; $idf\left(x\right)$ calculates how many instances contain the feature x; N represents how many ransomware samples in the corpus.

The study conducted by Chen and Bridges [43] used the TF-IDF for behavioral features extraction, by which the model identified the important features that represent the anomalous aspects in the crypto ransomware behavior. In addition, Nissim, Lapidot [33] utilized TF-IDF to represent the system calls extracted from crypto ransomware runtime data as numerical features suitable for various detection algorithms.

## 6. Features Selection Techniques

A major problem in malicious program detection models is the high dimensional features extracted during the features extraction phase [71,72]. In other words, the number of variables retrieved by N-gram amplifies with the number of N, and, as a result, the detection models become more susceptible to overfitting [38,55,56,69]. It is also worth noting that many of these features are either too widespread or too particular, making their knowledge of the attacks ineffective. Furthermore, a significant number of those characteristics are redundant and strongly associated [29,70,71]. This is due to the API calls’ reliance, which causes them to always be executed together [29]. As such, it is impractical to build the crypto ransomware detection model based on the entire features [58]. Hence, feature selection is a crucial step commonly used in building detection models to address the redundancy problem and avoid the curse of dimensionality [73]. 

To address the feature redundancy problem and decrease data dimensionality, the feature selection process is normally conducted before the model training [58]. Feature selection is the process of obtaining an appropriate subset of relevant and informative features that allows the detection model to detect the malicious attacks accurately and efficiently [58,74]. As such, many redundant and noise features are filtered out, which increases the detection accuracy [75]. Moreover, by decreasing feature dimensionality, the risk that the detection model could run into overfitting is decreased [42,76]. Several feature selection techniques, such as similarity-based, statistical-based, sparse-learning-based, and information theory-based techniques, are usually used in machine learning-based classification tasks including crypto ransomware early detection [42,57,58,62,70,77,78,79].

Similarity-based feature selection techniques assess the feature’s importance based on the ability to preserve data similarity. Amongst these techniques are Fisher Score [80], Laplacian Score [81], and Trace Ratio Criterion [82]. Despite the ability to perform well in both supervised and unsupervised learning problems, similarity-based feature selection techniques cannot handle feature redundancy, which leads to include many highly correlated features and, consequently, degrades the accuracy of each iteration of detection models [57].

Sparse learning-based feature selection techniques embed the selection process into the learning algorithm. As such, these techniques are optimized for the underlying learning algorithm. Efficient and Robust Feature Selection (ERFS) [83], Multi-Cluster Feature Selection [84], and $L_{1,2}$ Norm Regularized Discriminative Feature Selection [85] are examples of this type of feature selection techniques. In addition to its computational complexity, the selected subsets built by sparse learning-based techniques perform well only on the learning algorithm they were optimized for and, consequently, may not achieve good accuracy on the other algorithms [57].

Statistical-based features selection techniques employ predefined statistical measures to build a subset of informative features. Such measures include chi-square [86], low variance, Gini Index, and T-score. Although statistical-based features selection techniques are featured by their efficiency in terms of computational complexity, they evaluate each feature individually and hence cannot handle feature redundancy [57]. 

Among many features selection techniques, information theory-based techniques are characterized by having no assumptions about the data distribution, in addition to the ability to effectively handle the relevance–redundancy trade-off, which makes it suitable to work for any kind of data [57]. Mutual Information Feature Selection (MIFS) [87], Joint Mutual Information (JMI) [88], and minimum Redundancy Maximum Relevance (mRMR) [89] are among the common information theoretic-based features selection techniques that have been used by several crypto-ransomware and malware detection tasks [32,42,58,79,90].

The mutual information MI criteria are defined as the degree of knowledge that two discrete variables have about each other [52]. This metric is computed as per Equation (2) as follows:

(2) $$MI\left(X;Y\right)=H\left(X\right)-H(X|Y)=\underset{y\epsilon Y}{\sum }\underset{x\epsilon X}{\sum }p\left(x,y\right)\log\frac{p\left(x,y\right)}{p\left(x\right)p\left(y\right)}$$

where $H\left(X\right)$ is the entropy of X; H(X|Y) is the conditional entropy of the variable X given $Y;\;p\left(x\right)$ and $p\left(y\right)$ are the marginal distribution of x and  y; and $p\left(x,y\right)$ is the joint distribution of x and y. The entropy $H\left(X\right)$ and conditional entropy $H\left(X|Y\right)$ are calculated according to Equations (3) and (4) as follows:

(3) $$H\left(X\right)=-\underset{x_{i}\in X}{\sum }p\left(x_{i}\right)\log\left(p\left(x_{i}\right)\right)$$

(4) $$H\left(X|Y\right)=-\underset{y_{j}\in Y}{\sum }p\left(y_{j}\right)\underset{x_{i}\in X}{\sum }p\left(x_{i}|y_{j}\right)\log\left(p\left(x_{i}|y_{j}\right)\right)$$

In their study, Brown, Pocock [91] proposed a unifying framework for information theoretic features selection by which several feature selection techniques were proposed, including Mutual Information Features Selection (MIFS), Information Gain (IG), Minimum Redundancy Maximum Relevance (mRMR), and Joint Mutual Information (JMI). 

Equation (5) represents the general formula of the framework (by linear combinations of Shannon information terms), according to Li, et al. [54] and Brown, et al. [88]:

(5) $$J\left(X_{k}\right)=I\left(X_{k};Y\right)-\beta \underset{X_{j}\epsilon S}{\sum }I\left(X_{j};X_{k}\right)+\gamma \underset{X_{j}\epsilon S}{\sum }I\left(X_{j};X_{k}|Y\right)\;\;$$

where $I\left(X_{k};Y\right)$ is the mutual information between the candidate feature $X_{k}$ and the class label Y; $I(X_{j};X_{k}|Y)$ is the conditional mutual information between the candidate feature $X_{k}$ and the feature $X_{j}$ in the selected set S given the class label Y; β and γ are parameters (coefficients) with values between 0 and 1. Equation (5) consists of two parts: the relevance term (6) and redundancy term (7). In addition, the redundancy term has two sub-terms: marginal redundancy (8) and conditional redundancy (9).

(6) $$I\left(X_{k};Y\right)\;\;\;\;\;\;\;\;\;$$

(7) $$\beta \underset{X_{j}\epsilon S}{\sum }I\left(X_{j};X_{k}\right)+\gamma \underset{X_{j}\epsilon S}{\sum }I\left(X_{j};X_{k}|Y\right)\;$$

(8) $$\beta \underset{X_{j}\epsilon S}{\sum }I\left(X_{j};X_{k}\right)\;\;\;$$

(9) $$\gamma \underset{X_{j}\epsilon S}{\sum }I\left(X_{j};X_{k}|Y\right)\;\;$$

## 7. Detection Techniques

The early detection of ransomware has been tackled by Rhode, Burnap [28], Homayoun, Dehghantanha [31], Homayoun, Dehghantanha [40], Sgandurra, Muñoz-González [42] who captured the runtime data during the initial phases of the attacks. Furthermore, they utilized a fixed time-based approach to define the early phase, such that all crypto-ransomware samples were run for the same time. Based on the extracted data, the detection models were trained. Those models can be categorized into single-algorithm-based models such as those proposed by Homayoun, Dehghantanha [31], Sgandurra, Muñoz-González [42] and ensemble-based models such as those proposed by Rhode, Burnap [28], Homayoun, Dehghantanha [40].

The single algorithm-based models are detection models whose decisions are taken by single machine learning algorithm (classifier). This classifier is normally trained by one dataset that contains a set of attack instances. In their study, Sgandurra, Muñoz-González [42] captured the runtime data for 30 s from the beginning of the execution. This data was used to train a logistic regression classifier. Similarly, Homayoun, Dehghantanha [31] used the early data extracted from all crypto ransomware instances during the first 10 s of their execution time to train two deep learning-based algorithms, Long Short Term Memory (LSTM) and Convolutional Neural Networks (CNN), for crypto ransomware early detection.

By comparison, ensemble-based detection combines several machine learning algorithms for the same task. Several ensemble learning-based detection solutions, such as those of Rhode, Burnap [28], Homayoun, Dehghantanha [40], were proposed to detect the ransomware early. The idea is that by combining decisions of several weak classifiers, the ensemble can make more accuracy detection [28,45,92,93,94,95]. This is attributed to the complementary nature of the ensemble’s base classifiers, which allows the introspection of the versatility of the dataset that contributes to increase the detection accuracy [45,46,47,48]. To maximize the detection accuracy of ensemble-based detection models, the ensemble’s components need to be diverse and individually accurate [96]. It was found that the diversity is one of the important characteristics that determine the ensemble’s accuracy [45]. 

## 8. Limitations of Existing Research in Ransomware Early Detection

**Figure 2:**
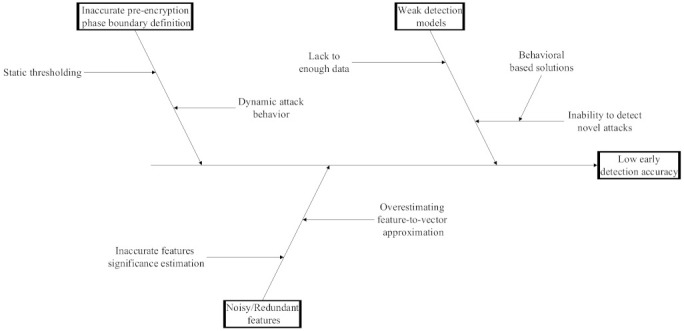
Cause–effect diagram illustrating the limitations in existing crypto-ransomware early detection solutions.

Crypto ransomware attacks are irreversible and resemble benign applications; detecting these attacks before they encrypt the data is critical. Although several solutions have been proposed to address the issue, those solutions suffer from several limitations that hinder their ability to detect such attacks effectively and accurately. These limitations are related to the inaccurate definition of the pre-encryption stage in the crypto ransomware lifespan, the insufficient data gathered during that stage, and the design of the detection model components, in a way that makes the approach unable to cope with such data limitations. The cause–effect diagram shown in Figure 2 summarizes those issues.

## 9. Limitations Related to Pre-Encryption Features Extraction

The idea of building machine learning-based detection models using the early data extracted during the onset of crypto ransomware attacks was introduced by Sgandurra, Muñoz-González [42]. To define the quantity of data required, the authors proposed fixed time-based thresholding by which the data captured during the first 30 s of the ransomware instances’ runtime were collected and used to train an ML classifier for the early detection. Similarly, Homayoun, Dehghantanha [40] and Homayoun, Dehghantanha [31] utilized the same approach, decreasing the threshold to 10 s, whereas Rhode, Burnap [28] decreased it to one second. However, the mentioned studies rely on a preset threshold value, which assumes all cases would not begin encrypting until the stated period has elapsed. This notion assumed in their work is incorrect. In many cases, the time it takes for the primary sabotage to begin differs across different cases due to the obfuscation methods used by these malicious programs, such as polymorphism and metamorphism, which produce distinct attack behavior among those instances [4,25,29,51,94]. Because of this, the fixed thresholding may miss the encryption starting point, leading to inaccurate detection of the pre-encryption phase of crypto ransomware attacks.

Despite the fact that Term Frequency-Inverse Document Frequency (TF-IDF) can compute attribute values more correctly than other approaches such as TF [29,95], the problem of implementing it to a limited percentage of the data emerges when computing the IDF term. This means that, while simply examining the pre-encryption data, a specific API may have a low Document Frequency (DF) value, but when analyzing the complete attack’s data, the API may have a large DF number. Thus, API pre-encrypted data have a high TF-IDF value, but API encrypted data have a low TF-IDF value. As a result, TF-IDF will incorrectly give that API more weight when depending only on pre-encryption data, even though it is a general-purpose API regarding all data and should be punished instead. This computation obstructs the ability of TF-IDF to provide an accurate numerical representation of the extracted features.

## 10. Limitations Related to Feature Selection

To ensure accurate early detection of ransomware, a relatively small quantity of data must be recorded during the malware’s first execution. This problem becomes more complicated within a high dimensional feature space and with many redundant features, making the model prone to overfitting [33,57,77,97]. The information theoretical-based feature selection techniques, such as MIFS, mRMR, and JMI, weigh the trade-off between relevance and redundant terms by adjusting their redundancy coefficients [57,91]. Moreover, those coefficients are adjusted to either a fixed or dynamic value [54,87,88,89,91].

Thus, selecting a fixed value for those parameters is difficult and needs to be set experimentally [54,91]. It is also important to note that the dynamic values of the coefficients are altered inversely proportionately to the number of features that have previously been chosen, which alters the confidence in the redundancy term at each iteration [91]. Although such calculations are able to make the trade-off between the relevancy and redundancy terms [57,91], the insufficient number of attack observations in the early data makes it harder to perceive the common characteristics of the already-selected features in the selected set and compare them with the characteristics of the candidate feature. As such, these techniques become incapable of accurately determining the redundancy term to estimate the features’ significance. In addition, the cumulative sum approach that MIFS and many other techniques employ for feature-to-vector approximation causes overestimating the feature significance which, consequently, leads to selection of redundant and irrelevant features [54,98].

## 11. Limitations Related to Detection Model Design

Building weak classifiers is another complication related to the lack of sufficient data about the attacks during the early phases [28,55]. The assumption that existing models make regarding the completeness of the attack patterns at detection time may not necessarily be true in the case of *early* detection. This follows because early detection takes place while the data are not fully captured and/or available. If not enough of the attacks’ pattern is available, the detection accuracy is degraded [55].

Although ensemble learning has the ability to make better decisions by combining multiple base classifiers [28,45,92,93,94,95], the accuracy of the ensemble depends on the trade-off between the accuracy of its base components (such as base classifiers) and the diversity between those components [96]. However, and like single algorithm-based models, the individual accuracies of the base estimators of the ensemble are low due to insufficient information about the attack at early phases, causing the accuracy of the overall ensemble to degrade [96]. In addition, bagging randomly divides the original dataset into subsets [93]. In this way, one or more ransomware families may not be sufficiently represented in some subsets. The random selection used to create multiple feature sets can repeat irrelevant, redundant and/or noisy features in one or more of these sets, which has a negative impact on the ensemble’s accuracy [99,100]. Although the irrelevant and noisy features in each subspace can be removed by conducting feature selection on each data subset, the selection will decrease diversity of the entire ensemble. Ironically, we know that some features are relevant and they are likely to occur in most or all subspaces. Consequently, the diversity of feature subsets will decrease, which also decreases the detection accuracy of the ensemble [101]. This is a severe problem for early detection.

For early detection of crypto ransomware, behavioral-based detection methods (also called misuse-based) have been developed that utilize the well-known attack patterns associated with crypto ransomware. This strategy fails when confronted with attacks whose patterns were previously unknown to the detection model. These are known as new or zero-day attacks. [102,103]. A detection solution that relies on anomaly detection is capable of detecting zero-day attacks, but the high incidence of false alarms makes it vulnerable to false positives. [104]. The issue of false alarms is exacerbated with the benign-like nature of crypto ransomware [105]. This is due to employing legitimate cryptography tools owned by the underlying system to carry out the attack and targeting the user-related files instead of system-critical files [24,42,50,105]. Therefore, the anomaly detection model cannot distinguish between the normal behavior and the crypto ransomware benign-like behaviors, which increases the rate of false alarms. Table 1 summarizes the limitations of existing crypto ransomware early detection solutions.

## 12. Discussion and Research Directions

Existing crypto ransomware early detection approaches depend on data acquired during the first stages of the attack’s lifespan. Although this reduces the time window for detection, there are several limitations related to the accurate definition of the pre-encryption phases and the limited amount of data collected during this short time, as discussed above. Such limitations have a negative impact on both feature selection and model design and must be addressed. To this end, this section elaborates those consequential research directions.

A dynamic pre-encryption boundary definition approach based on the cryptography-related API calls is needed to accurately track the encryption starting point of each instance and extract the pre-encryption data that accurately represent this phase of the attack. Based on these data, the features could be extracted that represent the pre-encryption phase of the attack while distinguishing general patterns that could be found in both benign and malicious programs. Moreover, the issue of inaccurate Inverse Document Frequency (IDF) calculation in the case of incomplete data needs to be addressed such that the underlying TF-IDF feature extraction is enabled to correctly calculate the weight of each extracted feature.

The lack of a dataset that contains the early behavioral patterns for ransomware is another area that the research community can explore. On the one hand, the challenge of obtaining labeled data that separates pre-encryption data from post-encryption data is the major factor that makes it difficult to model the early behavior of ransomware. On the other hand, the correlation between pre-encryption and post-encryption behavior is very small, which makes the problem severe (i.e., difficult to separate either with a high level of confidence). This is partially due to the obfuscation strategies employed by the malware’s deliberate (i.e., determinant) behavioral changes to evade detection. Therefore, a prudent data acquisition strategy that considers such unique aspects is important.

To improve the estimate of feature importance in the absence of sufficient evidence regarding the behavior of crypto ransomware during the early (pre-encryption) stages of the assaults’ lifecycles, an improved trade-off between relevance and redundancy of the existing mutual information attribute selection approach is required. In addition, there is a need to address the feature relevancy overestimation by employing the maximum of minimum technique for feature-to-vector approximation. Both techniques could be incorporated into the mutual information feature selection, which could improve the accuracy of the model using the selected feature set.

The weak design of the detection model could be addressed by compensating for the data limitation and enhancing the ability to detect novel attacks. To compensate for data insufficiency at the early phases of crypto ransomware attacks, an incremental approach could be employed to derive several data subsets, each of which is used to train one base classifier in an ensemble-based model. The benefit of employing the incremental approach is that it builds the subsets to reflect the progression of the attacks at its different phases. As such, at any phase, there will be one or more base classifiers trained with enough patterns related to the current phase and able to make accurate decisions. Moreover, the anomaly approach could be integrated with the behavioral-based detection model to enhance the ability of the model to detect novel attacks and, consequently, increase the detection accuracy of the entire model.

## 13. Conclusions

In this paper, the issues related to the early detection of ransomware were discussed. The techniques relevant to the different phases of detection solutions have been explored. The existing works to improve feature extraction, selection, and behavioral modeling have been elaborated. The major issue with early detection is the lack of enough data and behavioral patterns at the beginning of the attack. Existing solutions follow a rigid approach by either defining a fixed boundary for the pre-encryption phase of the ransomware attack, or relying on a pre-defined list of cryptography-related APIs. Both approaches have a negative impact on the data relevancy to the attacks’ early behavior. Innovative approaches and solutions are still needed, especially those that investigate the possibility of incorporating data captured from different sources and processes to support and enrich the behavioral artefacts of malicious software. This could be achieved by investigating the correlation between the API and other events related to the malicious process.

## Figures and Tables

**Table 1:** Limitations of existing crypto ransomware early detection solutions.

Authors	Technique			Limitation
	Feature Extraction	Feature Selection	Training/Detection	
Sgandurra, Muñoz-González [42]	Static threshold (30 s) Bag of Words and Term Frequency (TF)	Mutual Information (MI).	Logistic Regression.	Static thresholding. Feature extraction technique treats all features (APIs) equally and does not distinguish the features related to the attack from the general purpose general ones due to incomplete attack data.
Homayoun, Dehghantanha [40]	Static threshold (10 s) Sequential Pattern Mining (SPM) with Maximal Frequent Pattern (MFP)	Single step transition MSP.	Decision Tree, Random Forest, Bagging, MLP.	Static thresholding. Applying SPM to an incomplete attack pattern might lead to extract suboptimal sequences, especially with polymorphic crypto-ransomware types that continuously change the execution sequence. As mentioned by Das, Liu [55], the sequential approach is not effective for malware detection as it may perform its actions in a different order.
Rhode, Burnap [28]	Static threshold (1 s) Performance counter metrics such as CPU, memory, etc.		Recurrent Neural Networks (RNN).	Static thresholding. Deep learning works well only when used with big data [30].
Homayoun, Dehghantanha [31]	Static threshold (10 s) Bag of Words and Term Frequency (TF), which is embedded in LSTM and CNN.	Excluding the features using pre-defined threshold at the embedding step.	Convolutional Neural Networks (CNN) and Long Short Term Memory (LSTM).	Static thresholding. Deep learning works well only when used with big data. TF treats the attack-specific and general purpose features equally.

## Data Availability

Not applicable. We did not report any data.
